# Endovascular Treatment of Ruptured Middle Cerebral Artery Aneurysms With a Low-Profile Visualized Intraluminal Support Device

**DOI:** 10.3389/fneur.2020.631745

**Published:** 2021-01-28

**Authors:** Gaici Xue, Yu Zhou, Peng Liu, Qiao Zuo, Pengfei Yang, Yibin Fang, Qiang Li, Rui Zhao, Yi Xu, Bo Hong, Qinghai Huang, Jianmin Liu

**Affiliations:** ^1^Department of Neurosurgery, General Hospital of Southern Theatre Command of PLA, Guangzhou, China; ^2^Department of Neurosurgery, Changhai Hospital, Second Military Medical University, Shanghai, China

**Keywords:** intracranial aneurism, middle cerebral artery, low-profile visualized intraluminal stent, ruptured, safety, vascular disorders

## Abstract

**Objective:** Stenting in ruptured middle cerebral artery (MCA) aneurysms was reported with a high perioperative complication rate. However, the treatment devices and physician's experience have continued to evolve. We performed this retrospective study to evaluate the safety and efficacy of LVIS stent-assisted coiling for ruptured MCA aneurysms.

**Methods:** Patients with acutely ruptured MCA aneurysms treated between November 2014 and October 2019 were retrospectively reviewed. Clinical and angiographic data of those treated with LVIS stents were collected from a prospectively maintained database.

**Results:** A total of 40 patients with 40 ruptured MCA aneurysms were enrolled, which comprised 26.3% (40/152) of all the ruptured MCA aneurysms at the same time. All stents were successfully deployed except for one (2.5%), which had a poor stent opening. Ischemic procedure-related complications were encountered in three patients (7.5%). One patient died of complications related to high-grade SAH on admission. Follow-up (mean 15.9 months) angiography was performed for 36 patients, which showed 33 (91.7%) aneurysms were completely occluded, 1 (2.8%) was improved, 1 (2.8%) was stable, and 1 (2.8%) was recanalized. Clinical follow-up (mean 29.6 months) was available for all survived patients, which showed 38 (95.0%) patients had favorable neurologic outcomes (mRS score 0–2), and 2 (5.0%) patients had poor neurologic outcomes.

**Conclusion:** The use of LVIS stents is feasible, safe, and effective with glycoprotein IIb/IIIa inhibitor for the treatment of ruptured MCA aneurysms in the acute setting. Prospective, multicenter studies with larger sample sizes are still required to further evaluate the safety and long-term efficacy.

## Introduction

Endovascular coiling of middle cerebral artery (MCA) aneurysms is still technically challenging because of the complex anatomy, which includes wide necks and the incorporation of important branches (1). The intracranial stent is one of the most used tools to manage such complex lesions, and it has been proven to be safe and effective in many complex aneurysms, including unruptured MCA aneurysms (2). But uncertainty still existed about stenting in ruptured MCA aneurysms, which makes the decision of endovascular treatment for such a kind of aneurysms doubtful.

However, it is notable that the safety of stenting in ruptured intracranial aneurysms (RIA) continued to improve in recent years with the advancement of treatment materials and enriched experience (3). The Low-profile Visualized Intraluminal Support (LVIS) device (Microvention, Tustin, California, USA) is a self-expanding braided stent designed for intracranial complex aneurysms (4). They offered higher metal coverage rates and provided better aneurysm neck coverage and side-branch protection. Several recent studies have indicated favorable clinical and angiographic outcomes for complex lesions treated with this device, including RIA (4–8). But there are still few reports on its usage for the treatment of ruptured MCA aneurysms. In this article, we retrospectively analyzed the clinical and angiographic data of the ruptured MCA aneurysms treated with LVIS stent-assisted coiling in the past 5 years and aimed at evaluating the safety and efficacy of stent-assisted coiling with updated materials and strategies.

## Materials and Methods

The local institutional review board approved the study protocol, and the requirement for written informed consent was waived given the retrospective nature of the analysis. Between November 2014 and October 2019, 152 patients with 152 ruptured MCA aneurysms were admitted to our institution.

In our center, a “coiling first” policy was adopted. All patients, except for those with massive intrasylvian or intracerebral hematoma requiring immediate surgical evacuation or a patient's preference to clipping, were considered for endovascular treatment and were treated emergently after admission. The primary treatment strategy for acute RIA was not to use the stent as possible. Simple coiling, micro-catheter-assisted coiling, double microcatheter technique, and balloon remodeling are techniques considered in priority. But for aneurysms with a wide neck or involving side branches that were difficult to treat with these techniques, stenting would be performed. There should also be no contradictions for antiplatelet therapy, such as an intracerebral hematoma, which may demand surgical evacuation or external ventricular drainage.

**Figure 1:**
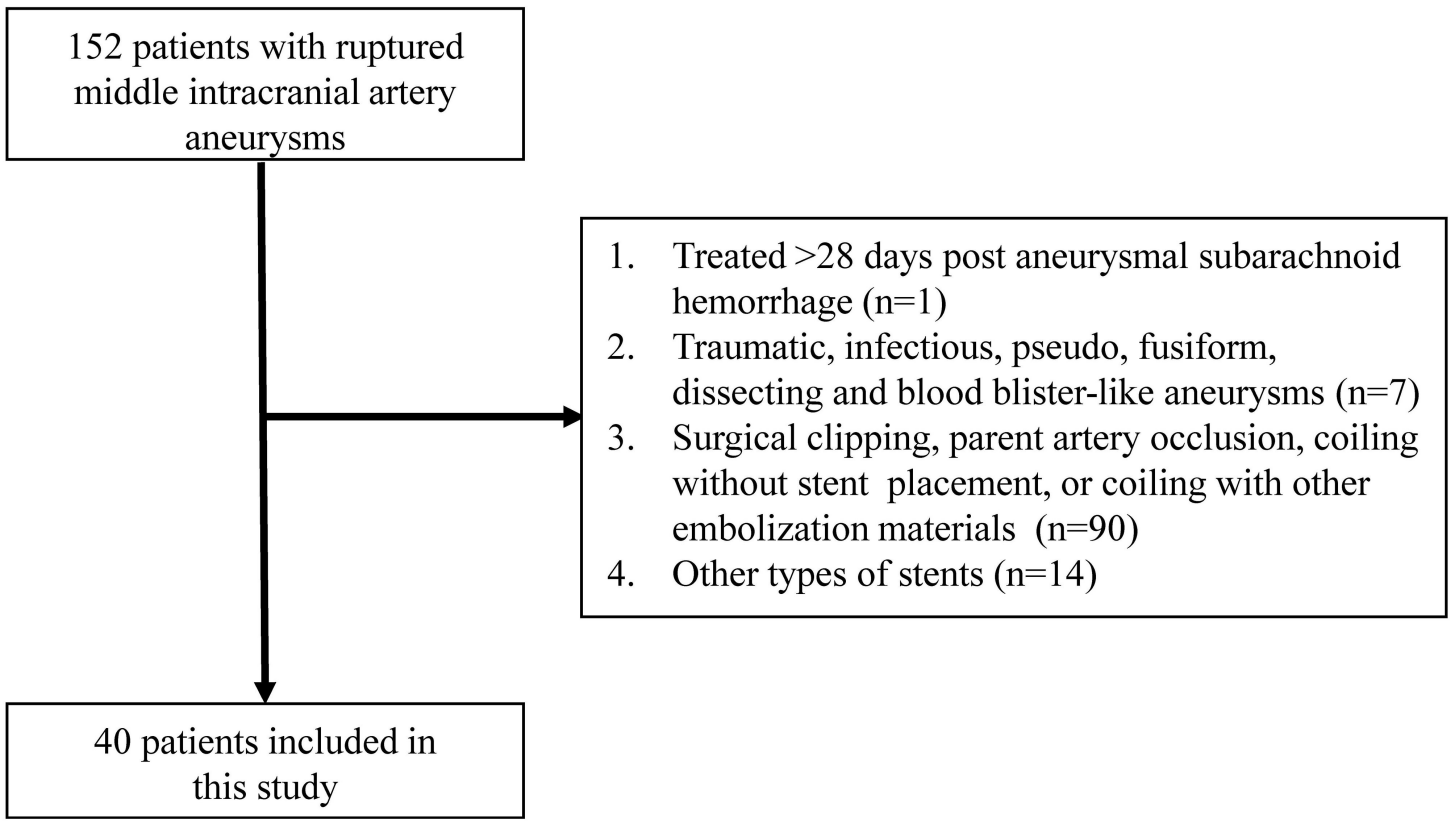
Flow diagram of patient selection according to the inclusion and exclusion criteria.

From a prospectively maintained database, we collected the clinical and angiographic data of those ruptured MCA saccular aneurysms treated with LVIS stent-assisted coiling, and excluded (a) those using stent as bailout technique and (b) treated >28 days post-aneurysmal subarachnoid hemorrhage for retrospective analysis (Figure 1).

### Endovascular Procedure

All procedures were performed with the patients under general anesthesia and via the transfemoral approach. After systemic heparinization, a 6F guiding catheter (Chaperon 6F; MicroVention, Columbia, California, USA) was placed in the distal internal carotid artery. All stents were deployed following the standard procedure recommended by the manufacturer. Stent placement was generally performed with modified jailing or semi-deployment technique (9).

The Echelon-10 (Covidien/ev3, Irvine, California, USA) microcatheter was initially positioned within the aneurysm and then we introduced one coil into the aneurysm sac to help stabilize the microcatheter; then the glycoprotein IIb/IIIa inhibitor (Tirofiban; Grand Pharma, Wuhan, China) infusion was initiated. After that, the stent was partially deployed to cover the aneurysm neck via a Headway microcatheter (Microvention/Terumo, Tustin, California, USA), and the aneurysm was packed with more coils. The coil microcatheter can be adjusted when necessary. After coiling, we released the stents into the parent artery. Generally, the Target coils (Stryker Neurovascular, Fremont, California, USA) were preferred for small and tiny aneurysms when stenting in intracranial aneurysms, and, when possible, hydrogel coils (Hydrogel coils; MicroVention Inc., Tustin, California, USA) would be introduced for these RIA due to its advantage of increasing the packing density. Dyna CT was routinely performed before and after each procedure to detect any aneurysm re-bleeding.

### Antiplatelet and Anticoagulation Therapy

All patients received systemic heparinization, and the activated clotting was maintained at 2–3 times the baseline throughout the procedure.

A loading dose of clopidogrel (300 mg) and aspirin (300 mg) was administered rectally or orally when deciding to perform coiling with stent placement. Simultaneously, a loading dose (5 μg/kg for 3 min) of tirofiban was administered intravenously before deploying the stent and followed by a maintenance of 0.075 μg/kg/min for 6 h. In the post-operative period, all patients were continued on aspirin (100 mg/d) and clopidogrel (75 mg/d) post-operatively for 6 weeks; this was followed by aspirin alone, which was maintained indefinitely. For patients who experienced thrombus formation during the procedure, a combined intra-arterial and intravenous administration of tirofiban would be conducted, but the total dose would not exceed the dose recommended by the instruction.

### Clinical and Angiographic Evaluation

Clinical outcome was obtained for all through the clinical evaluation at 3, 6, and 12 months after the treatment or via a telephone interview with the patient. Post-operative angiographic follow-up was recommended, including 3-month magnetic resonance angiography, 6-month digital subtraction angiography, and magnetic resonance angiography or digital subtraction angiography yearly thereafter. Immediate embolization results were evaluated according to Raymond-Roy occlusion classification and the follow-up results were classified into four categories when compared with the immediate embolization results: (1) occluded, defined as no contrast filling into the aneurysm sac; (2) improved, defined as decreased contrast filling into the aneurysm sac; (3) stable, defined as unchanged contrast filling into the aneurysm sac; and (4) recanalized, defined as increased contrast filling into the aneurysm sac (3).

### Statistical Analysis

Statistical analyses were performed using SPSS version 21.0 software (IBM Corp, Armonk, New York, USA). Categoric and continuous variables were presented as frequency and mean ± standard deviation, respectively.

## Results

### Patient Enrollment and Baseline Characteristics

In the study period, 152 patients with 152 ruptured MCA aneurysms were treated in our institution. Among these patients, 54 patients with 54 ruptured MCA saccular aneurysms were treated with stent-assisted coiling. Of them, 14 were excluded from this study because of using other types of stents (laser-cutting stents) due to the physician's discretion. Finally, 40 patients with 40 ruptured MCA aneurysms were included in this series.

As shown in Table 1, of these 40 patients, 17 (42.5%) were men and 23 (57.5%) were women. The mean age was 57.7 ± 10.4 (range 36–83) years, and the mean aneurysm size was 5.0 ± 2.4 mm. A total of 3 (7.5%) were located at the M1 segment, 36 (90.0%) were located at the bifurcation, and 1 (2.5%) was located at the M2 segment. Of the aneurysms, 26 (65.0%) were on the right side, and 14 (35.0%) were on the left side. The Hunt-Hess scale, when admitted, was grade I in 19 (47.5%), grade II in 18 (45.0%), and grade III in 3 (7.5%), respectively. Thirty patients (75.0%) were treated within 3 days of the aneurysm rupture, while the other 10 patients (25.0%) were treated beyond 3 days after aneurysm rupture because they were transferred to our institution with a substantial delay. Tirofiban was initiated before stent deployment in all patients save two cases in which, failing to follow the protocol, the antiplatelet regimen was administrated after stent deployment.

**Table 1:** Summary of the demographics and aneurysm characteristics.

**Variable**	**Value**
Total patients	40 (100)
Age, years	57.7 ± 10.4
Female	23 (57.5)
Size, millimeter	
Aneurysm size	5.0 ± 2.4
Neck size	3.4 ± 1.7
Hypertension	22 (55.0)
Diabetes mellitus	6 (15.0)
Coronary heart disease	1 (2.5)
Smoking history	6 (15.0)
Multiple aneurysms	7 (17.5)
Locatio	
M1	3 (7.5)
Bifurcation	36 (90.0)
M2	1 (2.5)
Hunt-Hess grade	
I	19 (47.5)
II	18 (45.0)
III	3 (7.5)
Modified fisher scale	
1	12 (30.0)
2	23 (57.5)
3	5 (12.5)
Interval between rupture and treatment	
≤ 1 day	20 (50.0)
1–2 days	4 (10.0)
2–3 days	6 (15.0)
3–4 days	5 (12.5)
4–5 days	5 (12.5)

*Data were presented as n (%) or mean ± standard deviation*.

### Immediate Embolization Results and Peri-Procedure Complications

**Figure 2:**
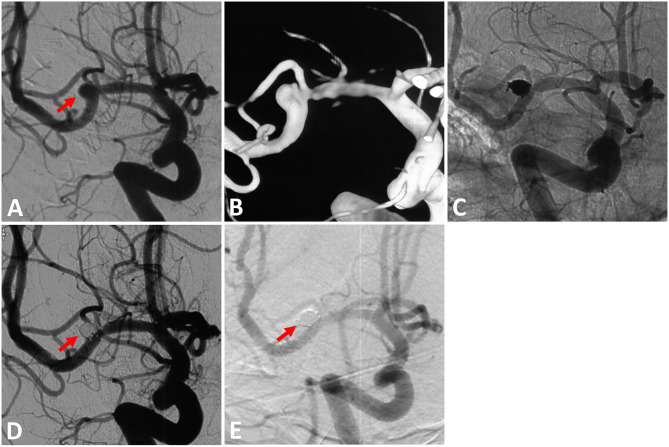
Illustrative case. **(A)** A 54-year-old woman with a 3.6 × 2.4 mm ruptured right middle cerebral artery (MCA) bifurcation aneurysm (solid arrow). **(B)** Three-dimensional reconstruction of the aneurysm. **(C)** The LVIS stent (3.5 mm × 15 mm) was successfully delivered in place. **(D)** Complete occlusion was achieved under the final view (solid arrow). **(E)** Digital subtraction angiography performed 16 months later showed complete aneurysm occlusion (solid arrow).

A total of 40 LVIS stents were implanted for these 40 ruptured MCA aneurysms (illustrative case in Figure 2). Post-operative angiograms showed complete occlusion in 23 (57.5%), neck remnant in 9 (22.5%), and the presence of the residual sac in 8 (20.0%).

Poor stent opening was observed in one patient in which the aneurysms harbored an acute angle between the parent and daughter artery. Fortunately, the artery was patent after prompt administration of intra-arterial tirofiban, and no neurological deficit occurred after the procedure.

There was no access complication. Ischemic complications occurred in 7.5% (3/40) of the patients, and no intra-operative rupture or other hemorrhagic complications occurred. Of these three patients, intra-stent thrombosis was observed in two patients who failed to follow the suggested antiplatelet protocol, in whom antiplatelet regimen was administrated after stents were partially deployed. But after combined intra-arterial and intravenous tirofiban infusion, no neurologic deficit events occurred in these two patients. Delayed procedure-related complications occurred in another patient. This patient experienced left hemiparesis (muscle strength grade III) 1 day after the procedure, and the CT scan showed massive cerebral infarction, intra-stent occlusion was suspected. Decompressive craniotomy was performed afterward. The patient remained hemiplegic at the 6-month follow-up.

### Clinical Outcomes

Except for these complications, three (7.5%) patients experienced symptomatic cerebral vasospasm during hospitalization, but all of them recovered well after aggressive anti-vasospasm treatments, which included intra-arterial fasudil (Chase Sun Pharma, Tianjin, China) infusion.

Clinical evaluation at discharge using the mRS showed 35 (87.5%) patients were independent with an mRS score of 0–2, whereas 5 (12.5%) patients were dependent. During clinical follow-up, 38 (95.0%) patients had favorable neurologic outcomes (mRS score 0–2), and 2 (5.0%) patients had poor neurologic outcomes within a mean period of 29.6 months (Table 2).

**Table 2:** Treatment outcomes and follow-up results.

**Variable**	**Value**
Immediate angiographic results	
Raymond class I	23 (57.5)
Raymond class II	9 (22.5)
Raymond class III	8 (20.0)
Procedure-related complications	3 (7.5)
Intraprocedural thrombosis	2 (5.0)
Post-procedural thrombosis	1 (2.5)
Technique complication	1 (2.5)
Symptomatic cerebral vasospasm	2 (5.0)
Procedure-related mortality	0 (0)
Clinical outcomes at discharge	
mRS score 0–2	35 (87.5)
mRS score 3	2 (5.0)
mRS score 4	3 (7.5)
Angiographic follow-up (mean, 15.9 months)	
Occluded	33(91.7)
Improved	1 (2.8)
Stable	1 (2.8)
Recanalized	1 (2.8)
Clinical follow-up (mean, 29.6 months)	
mRS score 0–2	38 (95.0)
mRS score 3	2 (5.0)

Data were presented as n (%) or mean ± standard deviation.

*mRS, modified Rankin scale*.

### Angiographic Follow-Up Results

Angiographic follow-up (mean, 15.9 months) was available for 36 (90.0%, 36/40) patients, and the angiographic results showed that 33 (91.7%, 33/36) aneurysms were completely occluded, 1 (2.8%, 1/36) was improved, 1 (2.8%, 1/36) was stable, and 1 (2.8%, 1/36) was recanalized (Table 2). The angiogram of the patient who experienced massive brain infarction after the procedure (described above in peri-procedure complication) showed parent artery occlusion, while no in-stent stenosis or parent artery occlusion was noted in other patients. The parent artery of the patient with poor stent opening was also patent.

## Discussion

This study shows that, with the current treatment technique and antiplatelet protocol, stenting in selected ruptured MCA aneurysms seems safe and effective, procedure-related morbidity and mortality were 7.5% and 0, respectively, and the complete occlusion rate reached 91.7% during follow-up.

Stenting in RIA in an acute phase is considered to have a high perioperative complication rate, which ranged from 8.3 to 27.7% in the previous studies (10–14). The incidence of perioperative complications was even higher when stenting in ruptured aneurysms located at small vessels beyond the circle of Willis (2, 15). Zhao et al. reported a retrospective study with 27 ruptured MCA aneurysms of stent-assisted coiling and noted that the incidence of acute intra-stent thrombosis was as high as 22.2% (6 of 27) (15). Our earlier study also showed that stent placement for acutely ruptured MCA aneurysms harbored a high procedure-related complication rate (25.9%, 7 of 27) (2). However, in this study containing a more recent population using updated materials and antiplatelet regimen, we observed a lower complication rate, only 7.5% of the patients experienced procedure-related ischemic events, and no hemorrhagic complications occurred.

Several factors may contribute to this improvement. First of these could be the advancement of treatment materials. Emerged softer coils, such as Target coils et al., may impose less radical force on the aneurysm sac, and hence reduce the risk of intraoperative aneurysm rupture. In addition, the availability of the braided LVIS stent also provided some advantages for the treatment of complex aneurysms. The “barrel technique” (pushing the stent across the aneurysm to make the stent partially protrude into the aneurysm) provides better protection for the wide aneurysm neck and the involved side-branches, and also enabled a better vessel attachment of the stent. A study comparing LVIS stent-assisted coiling (53 cases) and non-LVIS stent-assisted coiling (49 cases) for the treatment of unruptured MCA aneurysms also showed that coiling with LVIS stents increased treatment safety (16). Secondly, a modified antiplatelet regimen could help. One concern following stenting in MCA aneurysms is the high risks of ischemic events, especially for LVIS stent with such high metal coverage. For this reason, when using LVIS to manage ruptured MCA aneurysms, we added intravenous administration of tirofiban, in addition to dual antiplatelet drugs, to prevent thrombosis. The safety and efficacy of tirofiban have been proved in the management of RIA in a series of studies (10, 17–20). Wang et al. compared tirofiban with a loading dose of clopidogrel for preventing thromboembolism in stent-assisted coiling of RIA and found that tirofiban significantly decreased the incidence of thromboembolic events compared to clopidogrel (3.91 vs. 13.21%, *P* = 0.043) and did not increase the risk of hemorrhage (2.34 vs. 5.66%, *P* = 0.360) (18). Our antiplatelet regimen is modified based on our experience and previous literature (12, 19), that's a half dose of the tirofiban suggested by the package insert combined with dual antiplatelet drugs. No hemorrhagic complication was observed, and ischemic events occurred in 7.5% of the patients following such protocol in this study. Comparatively, this complication rate is significantly lower than that in our previous study (2). In that study, we used laser cutting stents to treat ruptured MCA aneurysms with the administration of a dual oral antiplatelet regimen, but the complication rate was as high as 25.9% (7/27), and 11.1% (3/27) were ischemic complications. Another concern for the usage of tirofiban was secondary hemorrhage caused by excessive antiplatelet therapy (21). Thereby, we always tried to embolize the aneurysm as densely as possible, and hydrocoils were always used to minimize the possibility of delayed aneurysm rupture (22). Moreover, there were no hemorrhagic complications in this series. The usage of tirofiban, combined with dual oral antiplatelet drugs, seemed to be safe and effective in our practice.

We also learned some lessons from our experience. First, the braided stent facilitated the embolization of the aneurysm. However, we should notice that such a stent harbors a lower radical force. In aneurysms harboring an acute angle between the parent and daughter artery, stent opening may be technically difficult. Second, the timing of administrating intravenous tirofiban also varies in different literature (10, 17, 20, 23). In our center, we usually used the semi-jailing or modified jailing technique to deploy the stent, so that we can adjust the coil microcatheter when necessary (9). In such situations, the tapered stent imposed higher metal coverage in the proximal segment of the parent artery. Tirofiban was therefore usually infused before the stent was delivered and deployed. As shown in our study, a delay of intravenous tirofiban may bring in higher complications, and two cases developed intra-stent thrombosis. In Kim's institution, they would initiate the tirofiban when contrast was no longer filling the dome of the aneurysm after partially coiling (10). But in some situations, it would be difficult; coils may protrude into the side-branches even when we introducing the first coil. We initiated the tirofiban after placement of the first coil, and no hemorrhage event was observed.

Endovascular treatment has been criticized for its lower complete occlusion and high recanalization rate during follow up for a long time. The previous series indicated a complete occlusion rate of 66.2–77.8%, a recanalization rate of 13.3–30%, and a re-treatment rate of 2.4–13.9% following the MCA aneurysm embolization (14, 15, 24–26). However, with the appliance of stent-assisted coiling, aneurysm angiographic outcome was improved, and the recanalization rate of MCA aneurysms also decreased to 4.9–7.1% (2, 3). Moreover, Samaniego et al. described a multicenter experience in treating eight MCA aneurysms with LVIS stents in a Y-stent configuration, and the follow-up angiographic results were excellent without aneurysm recurrence (27). In this present study of LVIS stent-assisted coiling for ruptured MCA aneurysms, 2.8% of the aneurysms recanalized, and complete occlusion increased to 91.7%. This favorable result resembled that of the TRAIL multicenter study, in which the complete occlusion rate was reported to be 92.4% at 18-month follow-up (8).

The limitations of this study included its retrospective design, the limited cases in one single institution, and the self-adjudication of clinical and angiographic outcomes. In addition, this is a small group of highly selective cases treated with a specific stent and a modified antiplatelet regimen; whether the conclusions of this study can be extended to other situations, such as other types of stents or other antiplatelet regimens, is in need of further study.

## Conclusions

The use of LVIS stents is feasible, safe, and effective with glycoprotein IIb/IIIa inhibitor for the treatment of ruptured MCA aneurysms in the acute setting with relatively low procedure-related complications rate and favorable long-term clinical and angiographic outcomes. Larger studies are still required to adequately assess the safety and long-term efficacy of this strategy.

## Data Availability Statement

The original contributions presented in the study are included in the article/supplementary material, further inquiries can be directed to the corresponding author/s.

## Ethics Statement

The studies involving human participants were reviewed and approved by Changhai Hospital's review board. Written informed consent for participation was not required for this study in accordance with the national legislation and the institutional requirements.

## Author Contributions

GX, QZ, PY, YF, QL, RZ, YX, BH, and QH contributed the conception, design, statistical analysis, and interpretation of data. GX, YZ, and PL collected the data and drafted the article. YZ and JL critically revised the manuscript. JL approved the final version of the manuscript on behalf of all authors.

## Conflict of Interest

The authors declare that the research was conducted in the absence of any commercial or financial relationships that could be construed as a potential conflict of interest.

## Abbreviations

MCA: middle cerebral artery
LVIS: Low-profile Visualized Intraluminal Support device
RIA: ruptured intracranial aneurysms.
